# The transcribed ultraconserved region *uc.160+* enhances processing and A‐to‐I editing of the *miR‐376* cluster: hypermethylation improves glioma prognosis

**DOI:** 10.1002/1878-0261.13121

**Published:** 2021-11-03

**Authors:** Marta Soler, Veronica Davalos, Anaís Sánchez‐Castillo, Carlos Mora‐Martinez, Fernando Setién, Edilene Siqueira, Manuel Castro de Moura, Manel Esteller, Sonia Guil

**Affiliations:** ^1^ Josep Carreras Leukaemia Research Institute (IJC) Barcelona Spain; ^2^ Department of Radiation Oncology (MAASTRO) GROW School for Oncology and Developmental Biology Maastricht University Medical Center The Netherlands; ^3^ Centre of Excellence in Experimental and Computational Developmental Biology Institute of Biotechnology University of Helsinki Finland; ^4^ Conselho Nacional de Desenvolvimento Cientifico e Tecnológico (CNPq) Brasilia Brazil; ^5^ Centro de Investigación Biomédica en Red Cáncer (CIBERONC) Madrid Spain; ^6^ Institució Catalana de Recerca i Estudis Avançats (ICREA) Barcelona Spain; ^7^ Physiological Sciences Department School of Medicine and Health Sciences University of Barcelona (UB) Spain; ^8^ Germans Trias i Pujol Health Science Research Institute Barcelona Spain

**Keywords:** A‐to‐I editing, glioma, miR‐376, noncoding RNA, pri‐miRNA biogenesis, T‐UCR

## Abstract

Transcribed ultraconserved regions (T‐UCRs) are noncoding RNAs derived from DNA sequences that are entirely conserved across species. Their expression is altered in many tumor types, and, although a role for T‐UCRs as regulators of gene expression has been proposed, their functions remain largely unknown. Herein, we describe the epigenetic silencing of the *uc.160+* T‐UCR in gliomas and mechanistically define a novel RNA–RNA regulatory network in which *uc.160+* modulates the biogenesis of several members of the *miR‐376* cluster. This includes the positive regulation of primary microRNA (pri‐miRNA) cleavage and an enhanced A‐to‐I editing on its mature sequence. As a consequence, the expression of *uc.160+* affects the downstream, *miR‐376*‐regulated genes, including the transcriptional coregulators RING1 and YY1‐binding protein (*RYBP*) and forkhead box P2 (*FOXP2*). Finally, we elucidate the clinical impact of our findings, showing that hypermethylation of the *uc.160+* CpG island is an independent prognostic factor associated with better overall survival in lower‐grade gliomas, highlighting the importance of T‐UCRs in cancer pathophysiology.

## Graphical abstract

The transcribed ultraconserved region *uc.160+* has been reported to be regulated by DNA methylation. Here, we describe that *uc.160+* increases the biogenesis of the *miR‐376* family through base‐pairing complementarity. The process includes enhanced pri‐miRNA processing and A‐to‐I editing. This represents a novel RNA–RNA regulatory network that acts in fine‐tuning the regulation of *miR‐376* targets such as RYBP and FOXP2, and has an impact on glioma prognosis. Our study highlights the potential use of *uc.160+* hypermethylation as a biomarker in patients with glioma.

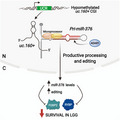

**Abbreviations**
ADAR: adenosine deaminase RNA specific
cDNA: complementary DNA
CGI: CpG Island
Chr: chromosome
CI: confidence interval
CRISPR: clustered regularly interspaced short palindromic repeats
EMSA: electrophoretic mobility shift assay
EV: empty vector
FOXP2: forkhead box P2
GBM: glioblastoma multiforme
Hsa: homo sapiens
Hg: human reference genome
HR: hazard ratio
HRP: horseradish peroxidase
IDH: isocitrate dehydrogenase
LGG: low‐grade glioma
miRNA/miR: microRNA
Mut: mutant
Ns: not significant
OS: overall survival
PCR: polymerase chain reaction
pre‐miR/pre‐miRNA: precursor microRNA
pri‐miR/pri‐miRNA: primary microRNA
RT: retrotranscription
RT‐qPCR: retrotranscription quantitative PCR
RYBP: RING1 and YY1‐binding protein
SCR: scramble
SD: standard deviation
sg/sgRNA: single guide/single guide RNA
TCGA: The Cancer Genome Atlas Program
T‐UCR: transcribed ultraconserved region
UCR: ultraconserved region
UCSC: University of California Santa Cruz
UTR: untranslated region
WT: wild‐type
χ^2^
: chi‐squared test

## 1 Introduction

Over the last decade, noncoding RNAs have been shown to play a variety of regulatory roles in gene expression networks, and thereby to have a broad influence on physiopathology [1]. Despite their heterogeneous origin and structure, one common feature is their relatively poor conservation across species [2]. However, there are some striking exceptions: The human genome contains several hundred ultraconserved regions (UCRs) that are 100% identical in human, mouse, and rat genomes, and are extensively transcribed, producing a class of long noncoding RNAs known as T‐UCRs [3, 4]. Most of these are expressed in a tissue‐specific manner in normal cells. Their functional relevance became evident with the identification of deregulated T‐UCR signatures associated with specific disease conditions [4, 5, 6, 7]. Moreover, altered profiles of T‐UCRs have been proposed as prognostic factors in human malignancies [8, 9].

Similar to other long noncoding RNAs, T‐UCRs may influence tumorigenesis by participating in proliferation, apoptosis, migration, or invasion, but little is known about their mode of action. For example, the *uc.454* T‐UCR increases apoptosis in lung cancer through direct interaction with the 3’UTR of *HSPA12B* mRNA [10]. *Uc.338* inhibits p21 signaling by interacting with the BMI1 polycomb family member [11], modulating the PI3K/AKT pathway [12], and negatively regulating TIMP‐1 3′UTR [13]. Importantly, many T‐UCRs are suspected of affecting miRNA abundance and function, mainly as a consequence of sequence complementarity between the two classes of RNAs [14, 15]. In a few cases, T‐UCRs have been shown to interfere with miRNA biogenesis through base‐pairing with the primary miRNA transcript [16, 17, 18], and likewise, T‐UCRs can be targeted by miRNAs. For example, overexpression of *miR‐155* in leukemia cells reduces the levels of *uc.160+* [4, 14, 19], and *miR‐153* suppresses *uc.416* expression in gastric cancer [19]. Based on microarray data, it was proposed that the T‐UCR signature in neuroblastoma prognosis groups is at least partially explained by the miRNA profile [14]. T‐UCR expression can also be modulated by epigenetic mechanisms. For instance, changes in local DNA methylation are associated with dysregulation of T‐UCRs in a variety of tumor types, and in some cases, are correlated with tumor stage. Hypermethylation of *uc.283+A*, *uc.160+,* and *uc.346+* CpG island is associated with silencing in cancer cells [7, 20]. Moreover, the cancer specificity of these methylation events highlights their potential as noninvasive biomarkers in circulating DNA, as recently shown in plasma samples of colorectal cancer patients [21].

We have previously described how miRNA biogenesis can be regulated by T‐UCRs: *uc.283+* controls pri‐miRNA processing through RNA:RNA complementarity with the lower stem region of the *pri‐miR‐195*, impairing miRNA biogenesis at the level of Drosha cleavage [16]. The biogenesis of miRNAs is a highly regulated process that can affect the amount and identity of mature miRNA [22]. Also, changes in the critical seed region of a miRNA (nucleotides 2–8 from the 5′ end of the miRNA) [23, 24] can reassign its specificity for target mRNAs. In the work reported here, we have identified *uc.160+* as an epigenetically regulated T‐UCR in human gliomas. Mechanistically, *uc.160+* regulates *miR‐376* cluster biogenesis through complementarity with the lower stem sequence of the pri‐miRNAs. This promotes Drosha cleavage and A‐to‐I editing of the mature miRNAs, with consequences for the regulated mRNA targets.

## 2 Materials and methods

### 2.1 Cell culture

Human glioma cell lines U‐87 MG and KS‐1 (purchased from the Japanese Collection of Research Bioresources Cell Bank) were maintained in Dulbecco's modified Eagle's medium (DMEM), supplemented with 10% v/v fetal bovine serum (FBS, Gibco, Waltham, MA, USA) at 37 °C in a humidified atmosphere of 5% CO_2_ and 95% air. Normal human astrocytes were purchased from Innoprot (#P10251). All cell lines were routinely checked for mycoplasma contamination.

### 2.2 Search for matches between pri‐microRNAs and T‐UCRs

Ultraconserved element sequences were downloaded from UCbase (www.ucbase.unimore.it) [25]; the strand from which they are transcribed is provided in ref. [4]. microRNA hairpin sequences were obtained from mirbase v.20 (www.mirbase.org) [26]. To find the matches between pairs of sequences, a regex‐based algorithm was implemented in Perl. The program compared every substring of a given minimum length (which was set to 11) from one database to another. When a match was found, adjacent nucleotides of the two sequences were sequentially compared with elongate it. Identical sequence matches between different hairpins of the same microRNA cluster and a T‐UCR were grouped accordingly.

### 2.3 Plasmid construction and generation of mutants by direct mutagenesis

The ultraconserved region within *uc.160+* was cloned from DNA into the pcDNA3.1(+) vector (Invitrogen), using primers that introduced the BamHI and EcoRV restriction sites for directional cloning. Pri‐miRNA sequences, including ˜ 150 bp upstream and downstream of the hairpins, were cloned from DNA into the pSPARK® TA vector (Canvax Biotech, Cordoba, Spain), with the sense orientation under T7 promoter, and expanded in *E. coli* DH5α bacteria. To generate *uc.160+* and pri‐miRNA mutants, overlapping primers that introduced the desired mutation were designed and used in PCRs with AccuPrime™ Pfx DNA Polymerase (Thermo Fisher Scientific, Waltham, MA, USA) to amplify the wild‐type plasmids. PCRs were then treated with 1 U of DpnI restriction enzyme (Takara) for 1.5 h at 37 °C to remove the parental plasmids. All oligos used are listed in Table S1.

### 2.4 Transient transfections

U‐87 MG and KS‐1 cells were transfected at 75–80% confluence with 8 µg of construct plasmids (pcDNA3.1‐uc.160+, pcDNA3.1‐uc.160+ mut12, or empty pcDNA3.1 vector as negative control) in a 100‐mm culture dish with jetPRIME® transfection reagent according to the manufacturer’s recommendations (1 : 2 DNA to jetPRIME® ratio (w/v)). Cells were harvested 48 h after transfection.

Synthetic mimics of *hsa‐miR‐376a‐3p* (MIMAT0000729), *hsa‐miR‐376c‐3p* (MIMAT0000720), and a negative control miRNA (scrambled, scr) were purchased from Shanghai GenePharma (Shanghai, China). The edited forms of *hsa‐miR‐376a‐3p* and *hsa‐miR‐376c‐3p* were purchased from Sigma‐Aldrich (St.Louis, MO, USA). Cells were plated onto a 100‐mm dish and transfected at 30–50% confluence with 40 nm of each miRNA mimic by using Lipofectamine 2000 transfection reagent (Thermo Fisher Scientific), according to the manufacturer’s protocols. Cells were harvested 48 h after transfection and analyzed in the following assays: RT‐qPCR, western blot, and editing. The oligonucleotides used are listed in Table S1.

MiRIDIAN® microRNA Hairpin Inhibitors (antagomiRs) against *hsa‐miR‐376a* (IH‐300683‐05‐0005), *hsa‐miR‐376c‐3p* (IH‐300674‐06‐0005), and a control scrambled antagomiR (miRIDIAN microRNA Hairpin Inhibitor Negative Control (IN‐001005‐01‐05)) were purchased from Dharmacon (Lafayette, CO, USA). AntagomiR transfections were performed at a final concentration of 200 nm (100 nm of Ant‐376a and 100 nm of Ant‐376c), using HiPerFect Transfection Reagent (Qiagen, Hilden, Germany), according to the manufacturer’s protocol. Cells were harvested 48 h after transfection and analyzed by western blot.

### 2.5 *miR‐376* cluster editing by CRISPR/Cas9

CRISPR/Cas9 technology was used for engineering *hsa‐miR‐376* cluster knockout in the KS‐1 cell line. Two sgRNAs (sgRNA1: 5′‐GCACTTTGCGAGTCCCACGT‐3′ and sgRNA2: 5′‐ATGGTGAGAGCAGCACACCG‐3′) were designed using the CRISPR Design tool page (http://crispr.mit.edu/) and cloned into the BbsI sites of pSpCas9 (BB)‐2A‐GFP (PX458) (#48138, Addgene®, Teddington, UK). Cells in a 100‐mm culture dish were transfected at 70% confluence with 8 µg (4 µg sgRNA1 and 4 µg sgRNA2) of construct plasmids with Lipofectamine™ Stem Transfection Reagent (Thermo Fisher Scientific). Two days after transfection, cells were detached and resuspended in 1X PBS, 2 mm EDTA, and 0.5% FBS, for flow cytometer selection. Cells containing green fluorescence (eGFP+) were selected by FACS and pooled. End‐point PCR was used to confirm the deletion of the target region. PCR primers are listed in Table S1.

### 2.6 Nuclear and cytoplasmic fractionation

Subcellular fractionation was performed with a PARIS™ kit (#AM1921, Life Technologies) as previously described [27]. Equal amounts of RNA from each fraction were subjected to RT‐qPCR, and the results were calculated using the comparative Ct method $2^{‐(\Delta C_{t})}$ and shown as a percentage, considering the total quantity of RNA recovered from each fraction. To verify the nuclear and cytoplasmic fractionation of the mRNA, *RNU6B* and *GAPDH* were used as controls, respectively. The separation was confirmed at the protein level by western blot with HISTONE H3 (#ab1791, Abcam, Cambridge, UK, 1 : 5000) and α‐TUBULIN HRP (#ab40742, Abcam, 1 : 5000).

### 2.7 RNA extraction and RT‐qPCR

Total RNA, including miRNAs, was extracted with a Promega Maxwell® RSC miRNA Tissue kit (AS1460, Promega, Madison, WI, USA) according to the manufacturer's recommendations. For expression analysis, total RNA was reverse‐transcribed using the RevertAid H minus Reverse Transcription Kit (EP0451, Thermo Fisher Scientific) with either oligodT primer (for mRNAs) or random primers (for T‐UCR). A negative control minus reverse transcriptase was run in parallel to control for genomic contamination. Real‐time PCRs were performed in triplicate in a QuantStudio™ 5 Real‐Time PCR system (Thermo Fisher Scientific), using 30–100 ng cDNA, 6 µL SYBR® Green PCR Master Mix (Thermo Fisher Scientific), and 416 nm primers in a final volume of 12 µL for 384‐well plates. All data were acquired and analyzed with QuantStudio™ Design & Analysis Software v1.3.1 and normalized with respect to the endogenous controls, *GUSB, PPIA,* and *HPRT1*. Relative RNA levels were calculated using the comparative Ct method $2^{‐(\Delta \Delta C_{t})}$. For miRNA expression analysis, the miRCURY LNA™ miRNA PCR assay system (Qiagen) was used, following the manufacturer’s recommendations, with the miRCURY LNA RT kit (Cat. No. 339340, Qiagen) for RNA retrotranscription, and the miRCURY LNA SYBR® Green PCR Kits (Cat. No. 339345, Qiagen) for the RT‐qPCR, in a QuantStudio™ 5 Real‐Time PCR (Thermo Fisher Scientific) apparatus with QuantStudio™ 5 software. To normalize the data, *RNU6B, miR‐191‐5p,* and *miR‐423‐3p* were used as the endogenous controls. *In vivo* pri‐miRNA processing was performed following total RNA extraction and reverse transcription with random primers and Superscript™ III Reverse Transcriptase (Cat. No. 18080044, Thermo Fisher Scientific). Oligos used for qPCR are listed in Table S1.

### 2.8 Western blot

Cell pellets were resuspended in Laemmli SDS sample buffer (10% glycerol, 2% SDS w/v, 63 mm Tris/HCl pH 6.8, 0.01% bromophenol blue) plus 2% 2‐mercaptoethanol, sonicated, and boiled for 5 min. Equal amounts of protein extracts were loaded onto Tris‐Glycine‐SDS gels and transferred to a nitrocellulose membrane (Whatman, GE Healthcare, Chicago, IL, USA), by liquid electroblotting (Mini Trans‐Blot Cell, Bio‐Rad, Hercules, CA, USA) for 1 h at 100 V. Membranes were blocked and incubated overnight at 4 °C with primary antibodies diluted in 5% nonfat milk in PBS containing 0.1% Tween‐20. The proteins detected were as follows: RYBP (#ab185971, Abcam, 1 : 1000), FOXP2 (#5335, Cell Signaling Technology, Denvers, MA, USA, 1 : 1000), LAMIN B1 (#ab16048, Abcam, 1 : 5000), α‐TUBULIN HRP (#ab40742, Abcam, 1 : 5000), HISTONE H3 (#ab1791, Abcam, 1 : 5000), ADAR1 (AMAB90535, Atlas Antibodies, 1 : 1000), and ADAR2 (HPA018277, Atlas Antibodies, Bromma, Sweden, 1 : 400). After three washes with PBS containing 0.1% Tween‐20, membranes were incubated for 1 h at RT in a bench‐top shaker with the secondary antibodies conjugated to horseradish peroxidase anti‐rabbit IgG (A0545, Sigma, 1 : 10 000) or anti‐mouse IgG (Na9310V, GE HealthCare, 1 : 5000). ECL reagents (Luminata‐HRT, Merck‐Millipore, Burlington, MA, USA, and SuperSignal West Femto, Thermo Fisher Scientific) and the iBright™ CL1500 Imaging System (Thermo Fisher Scientific) were used to visualize the proteins.

### 2.9 *In vitro* pri‐miRNA processing assay

*Pri‐miR‐376* RNA substrates for *in vitro* processing assays were prepared from DNA templates by standard *in vitro* transcription with T7 RNA Polymerase (Roche) in the presence of [α‐^32^P]‐ATP (PerkinElmer), as previously described [16]. RNA substrates corresponding to the ultraconserved region of *uc.160+* (322 nucleotides) were obtained by *in vitro* transcription from linearized DNA templates. Processing reactions were carried out with total HEK293T extracts, as previously described [28]. Briefly, each pri‐miRNA was incubated with increasing amounts (0.071, 0.155, and 0.284 μm) of *uc.160+*. The RNA mixture was preheated at 65 °C for 2 min and then cooled to 30 °C. The nuclear extract was added, and the reaction mixture incubated for a further 90 min at 30 °C. RNAs were phenol‐extracted, precipitated, and loaded onto an 8% denaturing polyacrylamide gel.

### 2.10 Determination of *pri‐miR‐376* cluster RNA editing sites

To measure the editing levels of *hsa‐miR‐376* family members, we followed the protocol previously described [29]. Total RNA was extracted from cell pellets using TRIzol® reagent (Invitrogen) and treated twice with DNase (#M6101, RQ1 RNase‐Free DNase, Promega). First‐strand cDNA was synthesized from 4 µg of total RNA with the SuperScript™ III Reverse Transcriptase (Cat. No. 18080044, Thermo Fisher Scientific), using two pri‐miRNA‐specific RT primers: the ‘pri‐miR376a2‐c editing Rv’ primer for *pri‐miRNA‐376a2* cluster and the ‘pri‐miR376a1‐b editing Rv’ primer for the *pri‐miRNA‐376a1‐b* cluster (Table S1). As a negative control, the same reactions were carried out with 4 µg of RNA without reverse transcriptase enzyme. cDNA products were then amplified with Immolase Taq polymerase (Bioline), using the specific forward and reverse PCR primers for each pri‐miRNA cDNA (Table S1). Products were isolated from agarose gel bands using a NucleoSpin® Gel and PCR Clean‐up (Macherey‐Nagel, Düren, Germany) and sequenced in a 3730 DNA Analyzer (Applied Biosystems). All RT‐PCR products were subcloned into the pGEM®‐T Easy Vector Systems (Promega), following the manufacturer’s protocol. For each cDNA, 75–100 clones were isolated and sequenced. Following analysis with bioedit v7.2.5 software, the frequency of editing was quantified as the ratio of the number of A‐to‐G changes to the total number of cDNA clones sequenced. A‐to‐I editing sites are indicated with a G in the chromatogram.

### 2.11 Electrophoretic mobility shift assay (EMSA)

The RNA substrates for *uc.160+*, *uc.160+ mut5,* and *uc.160+ mut12* were obtained and biotin‐labeled during the *in vitro* transcription by using 0.25 mm biotin‐16‐UTP (Roche) in the transcription reaction. Wild‐type or mut5 pri‐miRNA substrates were also synthesized by *in vitro* transcription from linearized DNA templates. Binding reactions were carried out in 1X binding buffer (20 mm Tris/HCl pH 8.0, 1 mm DTT, 1 mm MgCl_2_, 20 mm KCl, 10 mm Na_2_HPO_4_‐NaH_2_PO_4_ pH 8.0) with the biotin‐labeled RNA alone (0.1 pmol) or in the presence of increasing amounts (0.5–4 pmol) of unlabeled T‐UCR or pri‐miRNAs, in a final volume of 15 µL. Each RNA mixture was preheated at 70 °C for 5 min, gradually cooled down to let the RNA regain its native structure, and then left at 30 °C for 20 min. All reactions were then immediately loaded onto a native 6% polyacrylamide gel (29 : 1 acrylamide:bis‐acrylamide), transferred to a nylon membrane, and developed using a BrightStar® BioDetect™ Nonisotopic Detection Kit system (Thermo Fisher Scientific).

### 2.12 Databases and statistical evaluation

miRBase (http://www.mirbase.org) and TargetScanHuman (http://www.targetScan.org) were used to predict binding sites for the *miR‐376* family, and miRDB (http://mirdb.org) for target custom prediction of the edited miRNA forms. DNA methylation data of lower‐grade glioma (LGG) and glioblastoma multiforme (GBM) cases were extracted from the TCGA Data Portal (https://portal.gdc.cancer.gov/). The glioma cohort included patients for which methylation on the *uc.160+* locus, *IDH1* mutation, and survival was available.

Methylation levels (*β*‐value) of CpG sites > 0.33 were considered to be hypermethylated. Graphs and statistical comparisons were obtained with the graphpad prism 9.0.0 (La Jolla, CA, USA) and IBM SPSS Statistics (Armonk, NY, USA) for Windows. We used the Kaplan–Meier method to analyze patient data to estimate survival. The log‐rank test was used to establish any differences between patient groups. Hazard ratios (HRs) from univariate Cox regressions were used to determine the association between clinicopathological features and overall survival (OS). Multivariate Cox proportional hazards regression was used to identify the independent variables associated with OS. Experimental groups were compared using Student’s unpaired‐sample test. For association between variables, such as editing frequency, we used chi‐squared contingency and Fisher’s exact tests. All statistical tests were two‐sided. Levels of significance were recognized as *P* < 0.05 (*), < 0.01 (**) and < 0.001 (***).

## 3 Results and Discussion

### 3.1 The ultraconserved transcript *uc.160+* is commonly methylated in glioma samples and cell lines

**Fig. 1:**
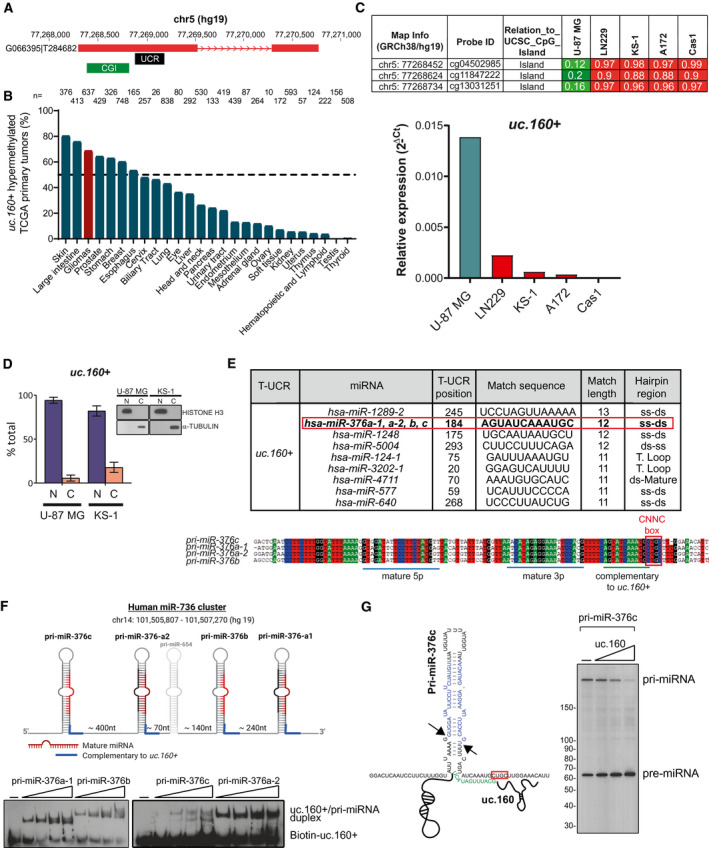
Methylation of the ultraconserved transcript *uc.160+* and complementarity with *miR‐376* family. (A) *uc.160+* genomic region and associated CpG island. Transcription of the RNA including the *uc.160+* ultraconserved region is supported by the annotated transcript G066395|T284682 in the MiTranscriptome database (mitranscriptome.org). *Uc.160+* coordinates are those given in the GRCh37/hg19 release. The black bar shows the sequence of *uc.160+* from the UCbase 2.0. database. The green bar shows the associated CpG island (CGI). (B) Percentage of *uc.160+* methylation in the TCGA panel of samples by tumor type. (C) *Above*, DNA methylation profile of the *uc.160+*‐associated CpG island in five glioma cell lines, analyzed by the 450K DNA methylation array in a previous study [69]. Single CpG absolute methylation levels (0–1) are shown. Green, unmethylated; red, methylated. *Below*, expression levels of *uc.160+* in the same cell lines as determined by real‐time PCR (*n* = 1). (D) Nuclear/cytoplasmic fractionation of U‐87 MG and KS‐1 cell lines, analyzed by RT‐qPCR and western blot to assess fraction purity. Graphs represent the mean ± SD of *n* = 2 replicates of fractionation. (E) *Table above*, Complementarity between *uc.160+* and pri‐miRNA hairpins, listed by match length. Only matches ≥ 11 nucleotides are shown; the region of complementarity is indicated. *Below*, hairpin regions of all pri‐miRNAs of the *miR‐376* cluster are aligned, and the region of complementarity to *uc.160+* is indicated. Results obtained with bioedit v7.0.5.3. (F) *Above*, diagram of the *mir‐376* cluster; nomenclature is from the miRBASE (http://microrna.sanger.ac.uk/sequences). Numbers at the bottom indicate intervening lengths of sequences. Created with Biorender.com. *Below*, electrophoretic mobility shift assay (EMSA) with biotin‐labeled *uc.160+* RNA and increasing levels of unlabeled *pri‐miRNA‐376a‐1/b/c* or *a‐2*. (0.5‐1‐2‐4 pmols). (G) *Left*, diagram to illustrate the region of complementarity (highlighted in green) between *uc.160+* and *pri‐mir‐376c* at the base of the stem, near the CNNC box (red box). The mature miRNA sequence is highlighted in blue. Hairpin structure is depicted according to [32]. Black arrows indicate Drosha cropping sites. *Right*, *in vitro* pri‐miRNA processing assays with ^32^P‐labeled *pri‐miR‐376c* in the presence of increasing amounts of *uc.160+*.

We have previously described that *uc.160+* undergoes cancer‐specific hypermethylation‐associated transcriptional silencing in some tumor types [20]. The full‐length transcript has been catalogued in the MiTranscriptome database of long polyadenylated RNA transcripts (www.mitranscriptome.org) with reference G066395|T284682, although it has not been yet annotated in Refseq (Fig. 1A). We first assessed the cancer‐specific hypermethylation of *uc.160+* by surveying normal and tumor samples from The Cancer Genome Atlas (TCGA) datasets, which revealed that > 50% of cases of most common primary tumor types (including gliomas, breast, and colon cancer) are hypermethylated (Fig. 1B, Table S2). By contrast, normal tissues show low or no methylation (Fig. S1A, Table S2).

We focused our study in gliomas, the most common, and lethal primary intracranial tumors. In accordance with the primary tumors, *uc.160+* was commonly hypermethylated in a panel of glioma cell lines but not in normal astrocytes (Table S2). Importantly, transcriptional silencing was observed in the hypermethylated cell lines, confirming the previously reported epigenetic regulation of *uc.160+* [20] (Fig. 1C). Analysis of U‐87 MG cDNA by RT‐PCR detected the MiTranscriptome‐annotated transcript, mostly in the unspliced form (Fig. S1B). In addition, cellular fractionation showed that *uc.160+* was enriched in the nucleus, as is the case for many unspliced transcripts that are deficiently exported (Fig. 1D), and *in silico* exploration of its coding potential drew attention to the transcript’s noncoding nature (Fig. S1C).

### 3.2 *uc.160+* is complementary to the *miR‐376* cluster and enhances its processing

Since our previous work and that of others have shown that some T‐UCRs regulate miRNA biogenesis through complementarity with their primary sequences, we next looked for potential complementarity (of at least 11 nucleotides) between *uc.160+* and miRNA primary sequences, excluding the mature regions (Fig. 1E). The longest complementarity (13 nucleotides) was found with *pri‐miR‐1289*, which is expressed at a very low level (mirbase.org, release 22.1) and for which no role in brain pathophysiology has been identified. Hits with 12 nucleotides included *miR‐1248* (also expressed at a very low level, according to the TissueAtlas, https://ccb‐web.cs.uni‐saarland.de/tissueatlas/ [30]), *miR‐5004* (expressed at a very low level and very poorly characterized), and the *miR‐376* family, which has the highest level of expression in the brain [30] (Fig. S2A), and whose lower level of expression in glioma predicts poor outcome [31]. These facts prompted us to investigate a possible interplay between *uc.160+* and the *miR‐376* family in gliomas. This family is transcribed as a cluster of pri‐miRNAs that include *pri‐miR‐376c*, *pri‐miR‐376a‐2*, *pri‐miR‐376b,* and *pri‐miR‐376a‐1*, from which the three most common mature *miR‐376s* are produced (all from 3p arms): *miR‐376c* (the most abundant), *miR‐376a,* and *miR‐376b* (Fig. 1E–F, Fig. S2A–B). All four pri‐miR‐376 hairpins have the 12‐nucleotide site complementary to *uc.160+* at the base of the stem‐loop structure, at the junction with the single‐stranded flanks (Fig. 1E–F). This region is within a stretch of 21 nucleotides that is identical in all pri‐miRNAs of the family, is even more conserved than the mature 5p or 3p miRNAs (Fig. 1E), and resides next to a ‘CNNC’ box (one structural motif on pri‐miRNAs that allows accurate processing through the recruitment of auxiliary factors [32, 33]). Direct binding between the pri‐miRNA sequences and *uc.160+* was confirmed by *in vitro* binding assays (Fig. 1F). We then generated the *uc.160+ mut12* and *uc.160+ mut5* substrates, in which 12 or 5 of the complementary nucleotides were mutated. In addition, compensatory mutations on each pri‐miRNA were also introduced to match *uc.160+ mut5* (Fig. S2C) (*uc.160+ mut12* could not be fully compensated on the pri‐miRNAs without potentially disrupting their hairpin structures). The use of these mutants in *in vitro* binding assays indicated that the complementary site was required for the interaction (Fig. S2D–E). Since miRNA biogenesis at the level of Drosha processing is tightly regulated, we next investigated whether this binding affected pri‐miRNA cleavage. Many protein factors are known to regulate this [33], but to our knowledge, only very few RNAs directly control miRNA biogenesis [16, 34, 35]. We ran *in vitro* processing assays with labeled pri‐miRNA sequences and confirmed that addition of the ultraconserved region of *uc.160+* enhanced cleavage and release of the pre‐miRNA. This is prominent with *pri‐miR‐376c*, which, in accordance with the high levels of *miR‐376c* found *in vivo*, is very efficiently processed *in vitro* (Fig. 1G), and is also seen with the other pri‐miRNAs from the *miR‐376* family (Fig. S2F–G). This regulation, together with the proximity of the CNNC box suggests that several factors, including ncRNAs, may converge around this 3’ flanking site to influence pri‐miRNA cleavage.

### 3.3 *uc.160+* enhances A‐to I editing of *miR‐376* family members

**Fig. 2:**
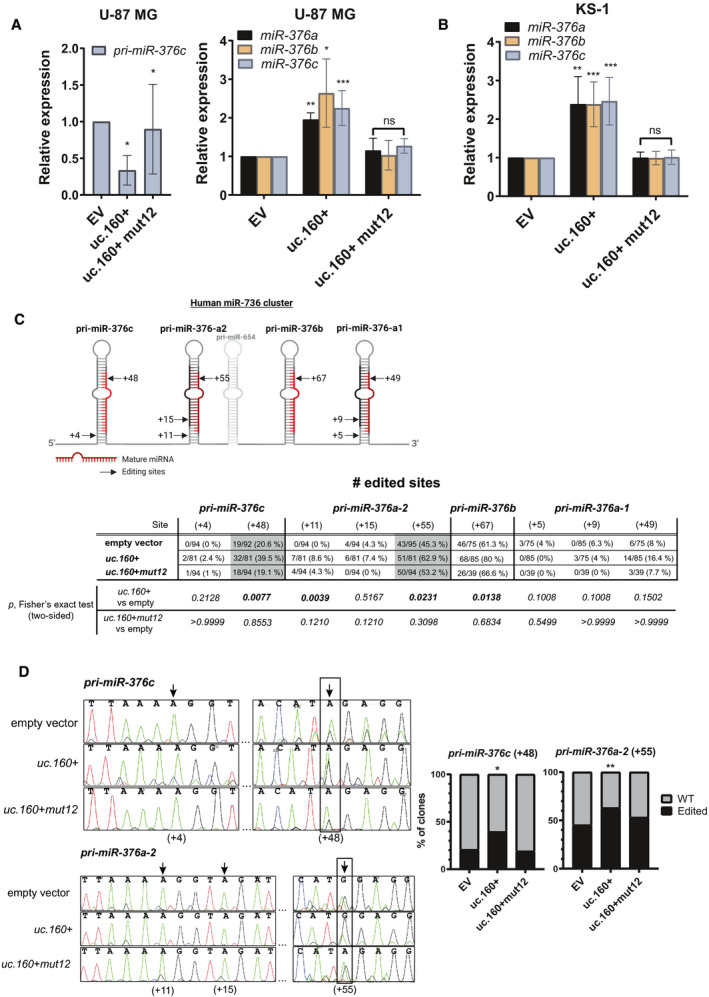
Influence of *uc.160+* on the processing and editing of the *miR‐376* family in glioma cell lines. (A, B) RT‐qPCR analysis of the endogenous levels of pri‐miR‐376c or mature *miR‐376a*, *miR‐376b,* and *miR‐376c* in U‐87 MG (A) and KS‐1 cell lines (B) transiently transfected with *uc.160+*, *uc.160+ mut12,* or empty vector (EV). Graphs represent the mean ± SD of three (A) or four (B) independent RNA extractions. Two‐tailed Student unpaired‐sample *t*‐tests were used to evaluate group differences (*P < 0.05, **P < 0.01, ***P < 0.001, ns = not significant). (C) Quantification of editing frequencies for *miR‐376* cluster editing sites in U‐87 MG cell line upon transient transfection of *uc.160+* or *uc.160+ mut12* vectors (empty vector was used as control). Sanger sequencing of RT‐PCR clones of primary miRNAs (pri‐miRs) from three independent experiments was employed. The increase in RNA editing in *pri‐miR‐376c* (position +48) and *pri‐miR‐376a‐2* (position +55) upon *uc.160+* overexpression is highlighted in gray. For each pri‐miRNA, the 5′ end of the stem‐loop sequence annotated in the Sanger miRBase dataset is counted as +1, as illustrated in the upper diagram of the cluster (created with Biorender.com). Editing frequency is calculated as the ratio of the number of A‐to‐G changes to the total number of cDNA clones sequenced. The statistical significance of the difference between empty vector and *uc.160+* or *uc.160+ mut12* overexpressed samples was assessed by chi‐squared contingency and Fisher’s exact tests. (D) *Left panel*: chromatograms of direct Sanger sequencing of RT‐PCR products corresponding to *pri‐miR‐376c* and *pri‐miR‐376a‐2* upon transient overexpression of *uc.160+* or *uc.160+ mut12*. A‐to‐I editing is detected as an A (green) to G (black) peak in the cDNA sequence. Black arrows indicate potential editing sites, and the most highly edited positions are boxed. *Right panel*: contingency graphs of the editing frequency of *pri‐miR‐376c* (position +48) and *pri‐miR‐376a‐2* (position +55), as identified in (C). The *y*‐axis shows the number of wild‐type (WT) and edited clones, as a percentage. Editing frequency was assessed by chi‐squared contingency and Fisher’s exact tests (*P < 0.05; **P < 0.01).

We next aimed to measure the impact of *uc.160+* on *pri‐miR‐376* processing in cells. In all glioma cell lines analyzed, endogenous *uc.160+* is expressed at low levels, so we assayed the impact of its ectopic expression. Overexpression in U‐87 MG and KS‐1 glioma cell lines resulted in a 2‐ to 3‐fold increase in the levels of mature *miR‐376a*, *b*, and *c*, confirming the ability of the T‐UCR to enhance *miR‐376* cluster processing (Fig. 2A–B). Moreover, this is accompanied by a concomitant reduction in *pri‐miR‐376c* levels (the only pri‐miRNA we could robustly detect by RT‐qPCR) and is abolished when the *uc.160+ mut12* was used, confirming that the complementary region is required for regulation (Fig. 2A–B). This prompted us to further analyze *miR‐376* biogenesis: In the brain, members of the *miR‐376* cluster are modified by hydrolytic deamination of adenosine to inosine (A‐to‐I editing) [36]. A‐to‐I editing is catalyzed by adenosine deaminases (ADARs), which require dsRNA for binding and editing, and is an important source of transcriptomic divergence from genomic DNA [37]. Inosines are functionally equivalent to guanosines, and therefore, A‐to‐I editing can directly affect the amino acid sequence of certain proteins when located on mRNA codons, as is the case for several neurotransmitter receptors and ion channels [38], thereby modulating neuronal signaling [39]. A‐to I editing is tightly regulated, and disruption of this process associates with neurological disorders and some types of cancer [40]. The systematic analysis of A‐to‐I editing in the TCGA datasets indicates that some nonsynonymous RNA editing events may be clinically relevant master driver events with crucial roles in cancer [41]. Most A‐to‐I substitutions are found on noncoding transcripts [42, 43], of which miRNAs are the best studied examples [44, 45, 46]. When A‐to‐I editing affects the seed region of a particular miRNA, it can modify target specificity and alter the profile of regulated mRNAs [47, 48]. miRNA editing can occur at the pri‐miRNA and pre‐miRNA levels, sometimes preventing the miRNA from maturing [49], or from loading onto RISC [50], or even leading to the degradation of the miRNA [51]. By contrast, previous studies have indicated that, in the case of *miR‐376* RNAs, editing does not affect the efficiency of the processing steps [36]. Given the link between editing and miRNA biogenesis, we next investigated whether the levels of editing of the *miR‐376* cluster were altered by *uc.160+* overexpression. Enforced expression of *uc.160+* (but not of *uc.160+ mut12*) in U‐87 MG cells significantly increased editing of *pri‐miR‐376c* (+48 site), *pri‐miR‐376a‐2* (+11 and +55 sites), and *pri‐miR‐376b* (+67 site), as measured by cDNA cloning and sequencing (Fig. 2C). Since *miR‐376b* is expressed at very low levels in U‐87 MG (Fig. S2B), we focused on *pri‐miR‐376c* and *pri‐miR‐376a‐2* and confirmed the hyperediting upon *uc.160+* overexpression by directly sequencing the cDNA (Fig. 2D). Position +11 on *pri‐miR‐376a‐2* is only marginally hyperedited and falls outside the mature 5p miRNA, and so was not further explored.

Conceptually, one way of regulating A‐to‐I editing is to alter the abundance of ADARs, but analysis by western blot revealed no changes in ADAR1 and ADAR2 protein levels upon T‐UCR overexpression, with varying levels of these enzymes being present in a panel of glioma cell lines (Fig. S3A–B). Rather, by analogy with how RNA‐binding proteins can alter editing in a site‐specific manner (e.g., by changing the dsRNA structure of the target [52, 53]), we can hypothesize that base‐pairing between *uc.160+* and pri‐miRNA transcripts can influence editing efficiency by promoting structural changes in the pri‐miRNA stem‐loop that enhances ADAR binding. Alternatively, this could be mediated by the Microprocessor itself, which acts as a recruiter of ADAR enzymes toward the target. In this second scenario, RNA:RNA interactions between T‐UCR and miRNA would favor Microprocessor recognition and, concomitantly, the recruitment of editing enzymes.

### 3.4 Identification of RYBP and FOXP2 as downstream targets

**Fig. 3:**
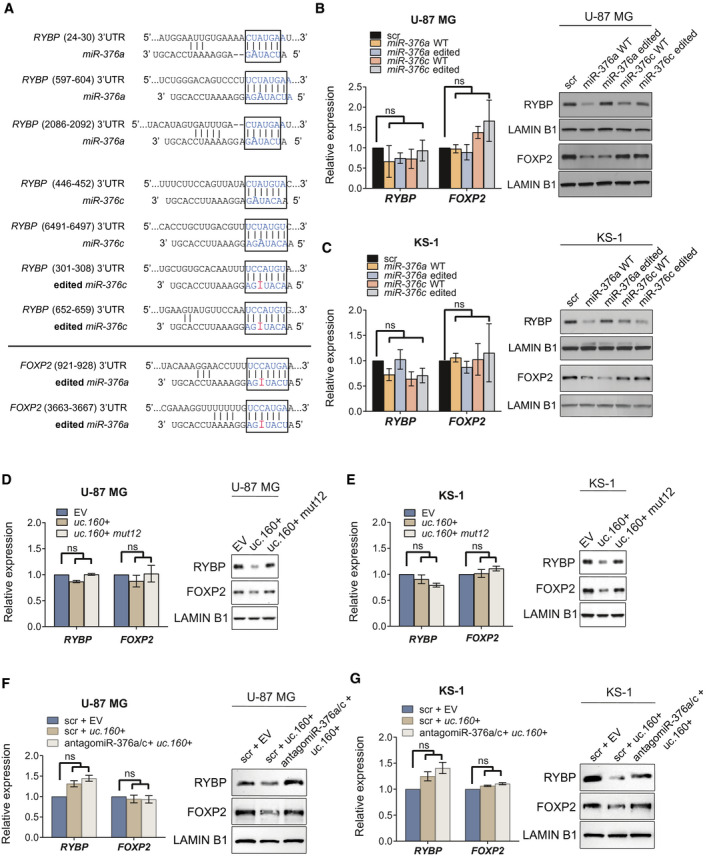
Impact of editing the *miR‐376* cluster on target regulation. (A) Predicted base‐pairing between the 3'UTR of *RYBP* (*above*) and *FOXP2* (*below*) mRNAs and the seed sequences of *miR‐376a‐3p* and *miR‐376c‐3p* (wild‐type and edited). The complementarity between the seed regions and target mRNAs is boxed. (B, C) Validation of RYBP and FOXP2 as *miR‐376* targets was achieved in U‐87 MG and KS‐1 cell lines following transient transfection of synthetic mimics of *hsa‐miR‐376a‐3p* or *hsa‐miR‐376c‐3p* wild‐type (WT), and their edited counterparts. Relative expression of endogenous mRNA levels was assessed by RT‐qPCR (*left*), and RYBP and FOXP2 proteins were analyzed by western blot (*right*). Graphs present the mean ± SD of three independent replicates. A Kruskal–Wallis test was used (ns = not significant). (D, E) Changes in RYBP and FOXP2 levels upon *uc.160+* or *uc.160+ mut12* overexpression in U‐87 MG and KS‐1 cell lines. Relative expression of endogenous mRNA levels was assessed by RT‐qPCR (*left*), and RYBP and FOXP2 proteins were analyzed by western blot (*right*). Graphs represent the mean ± SD of three independent experiments. One‐way ANOVA was used (ns = not significant). (F, G) Changes in RYBP and FOXP2 levels upon *uc.160+* overexpression in the presence of antagomiRs against *miR‐376a* and *c* in U‐87 MG and KS‐1 cell lines. Relative expression of endogenous mRNA levels was assessed by RT‐qPCR (*left*), and RYBP and FOXP2 proteins were analyzed by western blot (*right*). Graphs represent the mean ± SD of three independent replicates. One‐way ANOVA was used (ns = not significant).

Previous studies have reported the impact of mature *miR‐376a/c‐5p* editing on mRNA target regulation [36, 54], but editing on the 3p arms, which, according to miRBase, gives rise to the most strongly expressed mature miRNAs, has not been investigated in detail. One study has highlighted the weak effect of A‐to‐I editing on miR*‐376a‐3p* in terms of changing target specificity [47], and we next investigated this in the glioma cell lines. Positions +48 on *pri‐miR‐376c* and +55 on *pri‐miR‐376a‐2* are both on the corresponding ‘seed’ regions of *miR‐376c‐3p* and *miR‐376a‐3p* (hereafter referred to as *miR‐376c* and *miR‐376a*), and so can confer altered selectivity of target repertoire. To explore the impact of *uc.160+‐*induced hyperediting of *miR‐376* on its ability to regulate downstream mRNA targets, we set out to identify *bona fide miR‐376a* and *miR‐376c* targets in glioma cell lines. Using the TargetScan and microT‐DS mRNA target prediction tools, we found two common candidate genes for *miR‐376a* among the top 20 hits from each database: the RING1 and YY1‐binding protein *RYBP*, and the single‐stranded nucleic acid‐binding protein *RBMS1*. According to the miRDB prediction database, *RYBP* is also a potential target of unedited and edited forms of *miR‐376c*. In addition, the member of the forkhead/winged‐helix family of transcription factors *FOXP2* is one of the top newly predicted targets for edited (but not unedited) *miR‐376a* (Fig. 3A). These findings prompted us to experimentally validate the regulation of *RYBP* and *FOXP2* by the *miR‐376* family. Duplex miRNA mimics were designed against the unedited and edited forms of *miR‐376a* and *miR‐376c* and were transfected in U‐87 MG or KS‐1 cells (Fig. 3B–C). Neither *RYBP* nor *FOXP2* mRNA levels were significantly altered when the mimics were overexpressed; by contrast, the encoded proteins were downregulated under particular conditions. Specifically, as predicted, RYBP was targeted in the two cell lines by wild‐type *miR‐376a* and *miR‐376c*, and by the edited *miR‐376c* (Fig. 3B–C). FOXP2 protein was downregulated by the unedited and the edited forms of *miR‐376a*, although the level of downregulation in KS‐1 cells was greater with the edited miRNA, as expected (Fig. 3B–C). Once confirmed that *RYBP* and *FOXP2* are *bona fide* target genes regulated by the *miR‐376* family whose degree of specificity differs depending on the levels of mature miRNA editing, we investigated the impact of *uc.160+* on the identified *miR‐376* targets. Overexpression of *uc.160+* in U‐87 MG and KS‐1 cells had a clearly negative effect on RYBP and FOXP2 protein levels while not altering their mRNA levels, whereas cells transfected with *uc.160+ mut12* remained unaltered (Fig. 3D–E). Furthermore, this effect was abolished when *uc.160+* was cotransfected with antagomiRs against *miR‐376a* and *c* (Fig. 3F–G, Fig. S3C), and was also suppressed in KS‐1 cells that had been engineered by means of CRISPR/Cas9 to knockout the *miR‐376* cluster (Fig. S3D–E). Altogether, these data suggest a translational control by the ultraconserved transcript that is likely to proceed via regulation of *miR‐376* processing and editing.

RYBP is canonically known as an epigenetic factor with ubiquitin binding activity that associates with Polycomb complexes, is required throughout development, and has important roles in apoptosis and cancer [55]. Remarkably, high levels of RYBP protein induce apoptosis only in tumor cells, a feature of interest for cancer therapy [56, 57, 58, 59]. In fact, a lower level of RYBP has been observed in a number of tumor types, including glioblastoma, than in nontumoral tissue [60]. On the other hand, FOXP2 encodes a transcription factor with critical roles in neural development and brain circuits controlling language acquisition. Although the link between FOXP2 and oncogenic features is still uncertain, it belongs to a genomic domain containing a cluster of genes (including the *MET* oncogene) that often experiences cancer‐associated epigenetic changes [61]. An analysis of FOXP2’s transcriptional targets suggests that it may regulate the expression of pro‐oncogenic and tumor suppressor genes [61]. In the brain, FOXP2 displays proneurogenic activities by enhancing differentiation of neural precursors and reducing proliferation [62], and its increased expression has been associated with a poorer clinical outcome in neuroblastoma [63]. Given the suggested involvement of RYBP and FOXP2 in tumorigenic processes, the proposed role of the *miR‐376* family as an important biomarker in gliomas [31], and our findings indicating the epigenetic silencing of *uc.160+* in glioma cell lines (Fig. 1C) and its hypermethylation in 70% of gliomas from the TCGA cohort (Fig. 1B), we hypothesized that the aberrant DNA methylation of *uc.160+* could influence glioma patients’ outcome via the altered regulation of *miR‐376* cluster and downstream target genes.

### 3.5 Methylation of the *uc.160+*‐associated CpG island is an independent prognosis factor in lower‐grade glioma

**Fig. 4:**
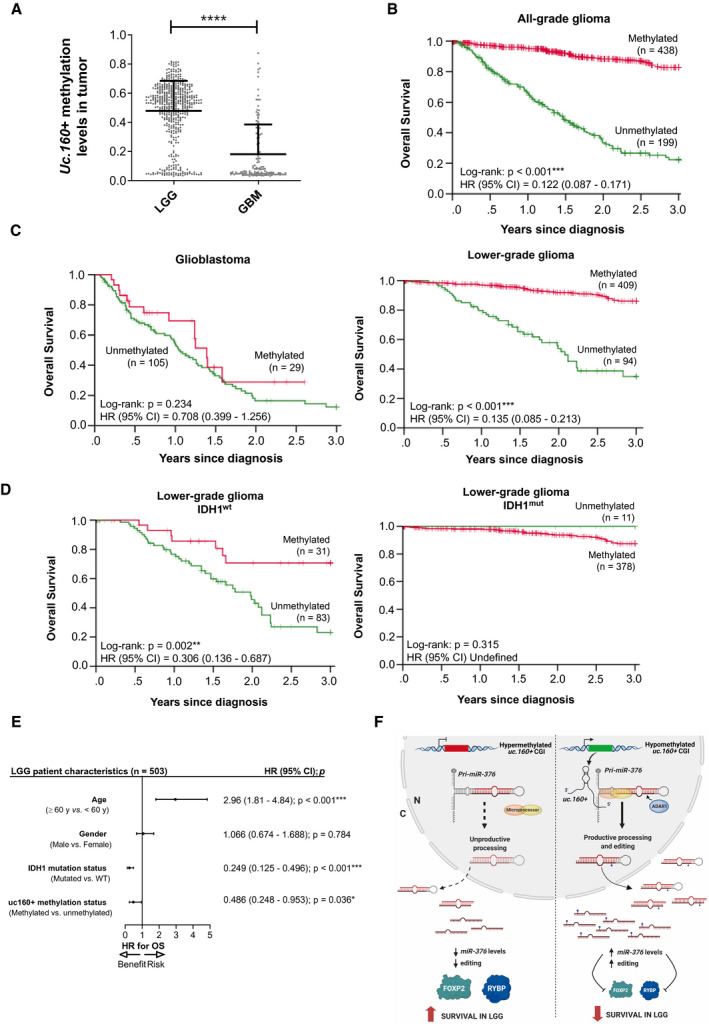
*uc.160+*‐CpG island methylation in human primary gliomas and its association with clinical outcome. (A) Levels of *uc.160+* CpG island methylation in the TCGA datasets of primary lower‐grade gliomas (LGG, *n* = 503) and high‐grade gliomas (glioblastoma multiforme, GBM, *n* = 134). An unpaired *t*‐test was used (****P < 0.0001). (B) Kaplan–Meier analysis of overall survival (OS) across all glioma grades from TCGA datasets with respect to *uc.160+* methylation status. (C) Kaplan–Meier analysis of OS in lower‐ and high‐grade glioma from TCGA datasets with respect to *uc.160+* methylation status. (D) Kaplan–Meier analysis of OS of lower‐grade glioma according to the molecular status of *IDH1* gene and *uc.160+* CpG island methylation levels. For all graphs in (B–D), the probabilities correspond to log‐rank tests. Results of univariate Cox regression are represented as the hazard ratio (HR) and 95% confidence interval (CI). (E) Forest plot of the multivariable Cox regression of clinical outcome in the TCGA lower‐grade glioma cohort on *uc.160+* methylation status. Probabilities (*P*) and 95% of confidence intervals (95% CI) correspond to the hazard ratios (HR) associated with OS. Significant covariates were considered independent prognostic factors (*P < 0.05; ***P < 0.001). (F) Summary of results. *Left*, CpG island hypermethylation‐associated epigenetic silencing of *uc.160+* compromises the efficient biogenesis of *miR‐376* family members and correlates with improved overall survival in lower‐grade glioma. *Right*, in hypomethylated samples, the expression of *uc.160+* increases processing and editing of *miR‐376*, modulating target regulation and correlating with poorer clinical outcome in LGG. Figure created with BioRender.com.

Gliomas are the most common and lethal type of intracranial tumors and have a very poor outcome and a median survival of 14–16 months (for grade IV gliomas). Since T‐UCR expression is not available for TCGA cohorts, we investigated changes in *uc.160+* promoter CpG island methylation, which we took to be a proxy of its expression in human primary gliomas. We analyzed the collections of lower‐grade gliomas (LGG) and glioblastomas (GBM) from the TCGA (https://portal.gdc.cancer.gov/). DNA methylation data were available for 503 LGGs, including 242 diffuse low‐grade and 261 intermediate‐grade gliomas (grades II and III, respectively, according to the World Health Organization (WHO) classification), and 134 GBMs (WHO grade IV) (Table S2). DNA hypermethylation of the *uc.160+* CpG island was detected in 68.75% of gliomas, with a clear enrichment in LGG (81.3% of methylated cases) in comparison with GBM (21.6% of methylated cases; Fisher’s exact test, *P* < 0.0001) (Fig. 4A). The decrease detected in *uc.160+* methylation as the disease progresses resembles other tumoral contexts: in colorectal cancer, a dynamic change in *uc.160+* methylation has also been observed, and methylation in stage III and IV patients has been associated with improved overall survival (OS) [21]. In addition, low methylation of *uc.160+* in GBM may be associated with the pro‐apoptotic role of RYBP in tumor cells [56, 57, 58, 59] and the worse predicted outcome when the level of RYBP is reduced [60]. Considering this, we next examined whether *uc.160+* methylation had any prognostic value in glioma patients. *Uc.160+* methylation was associated with increased OS when gliomas of all grades were analyzed together (log‐rank test: *P* < 0.001; hazard ratio (HR) = 0.122, 95% CI = 0.087–0.171) (Fig. 4B); however, considering the enrichment of LGG cases (*n* = 503) over GBM cases (*n* = 134), the weight of the 409 methylated LGG cases could bias the analysis. Independent analysis of LGG and GBM patients demonstrated that even though methylation is not able to stratify patients with dismal prognosis as glioblastomas (log‐rank: *P* = 0.234; HR = 0.708, 95% CI = 0.399–1.256), *uc.160+* CpG island methylation was significantly associated with increased OS in the lower‐grade gliomas (log‐rank: *P* < 0.001; HR = 0.135, 95% CI = 0.085–0.213) (Fig. 4C). We then examined whether *uc.160+* methylation helped define patient survival when combined with a genetic alteration of well‐recognized clinical impact in gliomas. Mutations in isocitrate dehydrogenase 1 (*IDH1*) are present in a high percentage of lower‐grade gliomas and, to a lesser extent, in high‐grade gliomas, and are markers of improved prognosis [64]. In glioblastoma samples, *uc.160+* methylation did not contribute to defining the survival of wild‐type *IDH1* patients (log‐rank: *P* = 0.991; HR = 1.003, 95% CI = 0.563–1.789) (Fig. S4A). By contrast, in the case of lower‐grade gliomas, although methylation of *uc.160+* did not contribute to patient stratification when *IDH1* was mutated (in which case the prognosis is generally good), wild‐type *IDH1* patients had better OS when *uc.160+* was hypermethylated (log‐rank: *P* = 0.002; HR = 0.306, 95% CI = 0.136–0.687) (Fig. 4D). Finally, multivariate Cox regression analysis including *IDH1* mutational status, age, and gender showed that *uc.160+* methylation is an independent prognosis factor for lower‐grade glioma patients (HR = 0.486; 95% CI = 0.248–0.953; *P* = 0.036) (Fig. 4E, Fig. S4B–C).

To summarize, our findings demonstrate that *uc.160+* is an epigenetically controlled T‐UCR with clinical relevance in gliomas. Mechanistically, our working model suggests that cells with high levels of *uc.160+* expression can process and edit *pri‐miR‐376* cluster more efficiently, with an effect on key protein targets such as RYBP and FOXP2 and, importantly, on lower‐grade glioma patients’ prognosis (Fig. 4F). Overall, our data highlight the impact of the regulatory roles of ultraconserved transcripts as fine‐tuners of other ncRNA biogenesis and their potential as biomarkers in the clinical practice. Approaches based on HITS‐CLIP have mapped globally the interaction between miRNAs and lncRNAs and have pointed to widespread cross‐regulatory mechanisms [65, 66], with an important impact on cellular physiology [67]. Given the complex, layered regulation that has been revealed for some T‐UCRs and miRNAs [68], we expect a bright future for these noncoding RNAs in translational settings. The discovery of additional features for pri‐miRNA recognition and processing and the process by which other ncRNAs influence their mature levels might lead to improved diagnostic and therapeutic tools in cancer and other diseases in which miRNAs are dysregulated.

## 4 Conclusions

The conclusions derived from this work can be summarized as follows:

- The transcribed ultraconserved region *uc.160+* displays a region of homology with *miR‐376* and enhances its production *in vitro* and in cell lines.
- This is accompanied by an increase in A‐to‐I editing on the mature *miR‐376*, and an impact on the downstream targets RYBP and FOXP2, which have roles in oncogenesis.
- Methylation of *uc.160+*‐associated CpG island in glioma patients helps define survival and is an independent factor for better prognosis in lower‐grade glioma.

## Conflict of interest

The authors declare no conflict of interest.

## Author contributions

All experiments were conceived by MS and SG and mainly carried out by MS. AS‐C optimized T‐UCR detection and analyzed its localization. CM‐M carried out the search for homology between T‐UCRs and miRNAs. VD and MCdeM performed the analysis of the clinical parameters. FS and ES designed and prepared genome‐edited cells. SG wrote the manuscript with the input of all authors. SG and ME supervised the project.

### Peer review

The peer review history for this article is available at https://publons.com/publon/10.1002/1878‐0261.13121.

## Supporting information


**Fig. S1.** Methylation status of *uc.160+* in normal tissues, expression patterns and coding petential.
**Fig. S2.** Expression of *miR‐376* cluster membres in tissues and cell lines, and *in vitro* modulation of their processing by *uc.160+*.
**Fig. S3.** ADAR1 and 2 levels in glioma cell lines, and disruption of *miR‐376* function.
**Fig. S4.** *uc.160+* CpG island hypermethylation in high‐grade gliomas and its association with clinical outcome.
**Table S1.** Oligos used in this work.

**Table S2.** DNA methylation data of 637 cases of glioma (503 LGG and 134 GBMs) available in TCGA (https://portal.gdc.cancer.gov/).

## Data Availability

The methylation data that support the findings of this study are available in Table S2.

## References

[1] Yao R‐W , Wang Y & Chen L‐L (2019) Cellular functions of long noncoding RNAs. Nat Cell Biol 21, 542–551. 31048766 10.1038/s41556-019-0311-8

[2] Derrien T , Johnson R , Bussotti G , Tanzer A , Djebali S , Tilgner H , Guernec G , Martin D , Merkel A , Knowles DG *et al*. (2012) The GENCODE v7 catalog of human long noncoding RNAs: analysis of their gene structure, evolution, and expression. Genome Res 22, 1775–1789. 22955988 10.1101/gr.132159.111 PMC3431493

[3] Bejerano G , Pheasant M , Makunin I , Stephen S , Kent WJ , Mattick JS & Haussler D (2004) Ultraconserved elements in the human genome. Science 304, 1321–1325. 15131266 10.1126/science.1098119

[4] Calin GA , Liu C‐G , Ferracin M , Hyslop T , Spizzo R , Sevignani C , Fabbri M , Cimmino A , Lee EJ , Wojcik SE *et al*. (2007) Ultraconserved regions encoding ncRNAs are altered in human leukemias and carcinomas. Cancer Cell 12, 215–229. 17785203 10.1016/j.ccr.2007.07.027

[5] Braconi C , Valeri N , Kogure T , Gasparini P , Huang N , Nuovo GJ , Terracciano L , Croce CM & Patel T (2011) Expression and functional role of a transcribed noncoding RNA with an ultraconserved element in hepatocellular carcinoma. Proc Natl Acad Sci USA 108, 786–791. 21187392 10.1073/pnas.1011098108 PMC3021052

[6] Carotenuto P , Fassan M , Pandolfo R , Lampis A , Vicentini C , Cascione L , Paulus‐Hock V , Boulter L , Guest R , Quagliata L *et al*. (2017) Wnt signalling modulates transcribed‐ultraconserved regions in hepatobiliary cancers. Gut 66, 1268–1277. 27618837 10.1136/gutjnl-2016-312278 PMC5530482

[7] Hudson Robert S , Yi M , Volfovsky N , Prueitt RL , Esposito D , Volinia S , Liu C‐G , Schetter AJ , Van Roosbroeck K , Stephens RM *et al*. (2013) Transcription signatures encoded by ultraconserved genomic regions in human prostate cancer. Mol Cancer 12, 13. 23409773 10.1186/1476-4598-12-13 PMC3626580

[8] Luo H‐L , Chen J , Luo T , Wu F‐X , Liu J‐J , Wang H‐F , Chen M , Li L‐Q & Li H (2017) Downregulation of macrophage‐derived T‐UCR uc.306 associates with poor prognosis in hepatocellular carcinoma. Cell Physiol Biochem 42, 1526–1539 28723685 10.1159/000479269

[9] Mestdagh P , Fredlund E , Pattyn F , Rihani A , Van Maerken T , Vermeulen J , Kumps C , Menten B , De Preter K , Schramm A *et al*. (2010) An integrative genomics screen uncovers ncRNA T‐UCR functions in neuroblastoma tumours. Oncogene 29, 3583–3592. 20383195 10.1038/onc.2010.106

[10] Zhou J , Wang C , Gong W , Wu Y , Xue H , Jiang Z & Shi M (2018) uc.454 inhibited growth by targeting heat shock protein family A member 12B in non‐small‐cell lung cancer. Mol Ther Nucleic Acids 12, 174–183. 30195756 10.1016/j.omtn.2018.05.004 PMC6023848

[11] Bo C , Li N , Li X , Liang X & An Y (2016) Long noncoding RNA uc.338 promotes cell proliferation through association with BMI1 in hepatocellular carcinoma. Hum Cell 29, 141–147. 27154519 10.1007/s13577-016-0140-z

[12] Zhang Y , Wang S , Qian W , Ji D , Wang Q , Zhang Z , Wang S , Ji B , Fu Z & Sun Y (2018) uc.338 targets p21 and cyclin D1 via PI3K/AKT pathway activation to promote cell proliferation in colorectal cancer. Oncol Rep. 10.3892/or.2018.6480 29901203

[13] Wang C , Wang Z , Zhou J , Liu S , Wu C , Huang C & Ding Y (2017) TUC.338 promotes invasion and metastasis in colorectal cancer. Int J Cancer 140, 1457–1464. 27914101 10.1002/ijc.30542

[14] Scaruffi P , Stigliani S , Moretti S , Coco S , De Vecchi C , Valdora F , Garaventa A , Bonassi S & Tonini GP (2009) Transcribed‐ultra conserved region expression is associated with outcome in high‐risk neuroblastoma. BMC Cancer 9, 441. 20003513 10.1186/1471-2407-9-441 PMC2804711

[15] Vannini I , Wise PM , Challagundla KB , Plousiou M , Raffini M , Bandini E , Fanini F , Paliaga G , Crawford M , Ferracin M *et al*. (2017) Transcribed ultraconserved region 339 promotes carcinogenesis by modulating tumor suppressor microRNAs. Nat Commun 8. 10.1038/s41467-017-01562-9 PMC5703849 29180617

[16] Liz J , Portela A , Soler M , Gómez A , Ling H , Michlewski G , Calin GA , Guil S & Esteller M (2014) Regulation of pri‐miRNA processing by a long noncoding RNA transcribed from an ultraconserved region. Mol Cell 55, 138–147. 24910097 10.1016/j.molcel.2014.05.005

[17] Guo J , Fang W , Sun L , Lu Y , Dou L , Huang X , Tang W , Yu L & Li J (2018) Ultraconserved element uc.372 drives hepatic lipid accumulation by suppressing miR‐195/miR4668 maturation. Nat Commun 9. 10.1038/s41467-018-03072-8 PMC5807361 29426937

[18] Xiao L , Wu J , Wang J‐Y , Chung HK , Kalakonda S , Rao JN , Gorospe M & Wang J‐Y (2018) Long noncoding RNA uc.173 promotes renewal of the intestinal mucosa by inducing degradation of microRNA 195. Gastroenterology 154, 599–611 29042220 10.1053/j.gastro.2017.10.009 PMC5811324

[19] Goto K , Ishikawa S , Honma R , Tanimoto K , Sakamoto N , Sentani K , Oue N , Teishima J , Matsubara A & Yasui W (2016) The transcribed‐ultraconserved regions in prostate and gastric cancer: DNA hypermethylation and microRNA‐associated regulation. Oncogene 35, 3598–3606. 26640143 10.1038/onc.2015.445

[20] Lujambio A , Portela A , Liz J , Melo SA , Rossi S , Spizzo R , Croce CM , Calin GA & Esteller M (2010) CpG island hypermethylation‐associated silencing of non‐coding RNAs transcribed from ultraconserved regions in human cancer. Oncogene 29, 6390–6401. 20802525 10.1038/onc.2010.361 PMC3007676

[21] Kottorou AE , Antonacopoulou AG , Dimitrakopoulos F‐ID , Georgia D , Chaido S , Melpomeni K , Theodoros T , Chrysa O , Katsakoulis EC , Angelos K *et al*. (2018) Deregulation of methylation of transcribed‐ultra conserved regions in colorectal cancer and their value for detection of adenomas and adenocarcinomas. Oncotarget 9, 21411–21428. 29765549 10.18632/oncotarget.25115 PMC5940382

[22] Michlewski G & Cáceres JF (2019) Post‐transcriptional control of miRNA biogenesis. RNA 25, 1–16. 30333195 10.1261/rna.068692.118 PMC6298569

[23] Lim LP , Lau NC , Garrett‐Engele P , Grimson A , Schelter JM , Castle J , Bartel DP , Linsley PS & Johnson JM (2005) Microarray analysis shows that some microRNAs downregulate large numbers of target mRNAs. Nature 433, 769–773. 15685193 10.1038/nature03315

[24] Lewis BP , Burge CB & Bartel DP (2005) Conserved seed pairing, often flanked by adenosines, indicates that thousands of human genes are microRNA targets. Cell 120, 15–20. 15652477 10.1016/j.cell.2004.12.035

[25] Taccioli C , Fabbri E , Visone R , Volinia S , Calin GA , Fong LY , Gambari R , Bottoni A , Acunzo M , Hagan J *et al*. (2009) UCbase & miRfunc: a database of ultraconserved sequences and microRNA function. Nucleic Acids Res 37: D41–D48. 18945703 10.1093/nar/gkn702 PMC2686429

[26] Griffiths‐Jones S (2004) The microRNA registry. Nucleic Acids Res 32, 109D–111. 10.1093/nar/gkh023 PMC308757 14681370

[27] Oliveira‐Mateos C , Sánchez‐Castillo A , Soler M , Obiols‐Guardia A , Piñeyro D , Boque‐Sastre R , Calleja‐Cervantes ME , Castro de Moura M , Martínez‐Cardús A , Rubio T *et al*. (2019) The transcribed pseudogene RPSAP52 enhances the oncofetal HMGA2‐IGF2BP2‐RAS axis through LIN28B‐dependent and independent let‐7 inhibition. Nat Commun 10. 10.1038/s41467-019-11910-6 PMC6726650 31484926

[28] Guil S & Cáceres JF (2007) The multifunctional RNA‐binding protein hnRNP A1 is required for processing of miR‐18a. Nat Struct Mol Biol 14, 591–596. 17558416 10.1038/nsmb1250

[29] Kawahara Y (2012) Quantification of adenosine‐to‐inosine editing of microRNAs using a conventional method. Nat Protoc 7, 1426–1437. 22743833 10.1038/nprot.2012.073

[30] Ludwig N , Leidinger P , Becker K , Backes C , Fehlmann T , Pallasch C , Rheinheimer S , Meder B , Stähler C , Meese E *et al*. (2016) Distribution of miRNA expression across human tissues. Nucleic Acids Res 44, 3865–3877. 26921406 10.1093/nar/gkw116 PMC4856985

[31] Huang Q , Wang C , Hou Z , Wang G , Lv J , Wang H , Yang J , Zhang Z & Zhang H (2017) Serum microRNA‐376 family as diagnostic and prognostic markers in human gliomas. Cancer Biomark 19, 137–144. 28211798 10.3233/CBM-160146 PMC13020711

[32] Auyeung VC , Igor U , McGeary SE & Bartel DP (2013) Beyond secondary structure: primary‐sequence determinants license Pri‐miRNA hairpins for processing. Cell 152, 844–858. 23415231 10.1016/j.cell.2013.01.031 PMC3707628

[33] Kim K , Nguyen TD , Li S & Nguyen TA (2018) SRSF3 recruits DROSHA to the basal junction of primary microRNAs. RNA 24, 892–898. 29615481 10.1261/rna.065862.118 PMC6004053

[34] Han K , Wang F‐W , Cao C‐H , Ling H , Chen J‐W , Chen R‐X , Feng Z‐H , Luo J , Jin X‐H , Duan J‐L *et al*. (2020) CircLONP2 enhances colorectal carcinoma invasion and metastasis through modulating the maturation and exosomal dissemination of microRNA‐17. Mol Cancer 19. 10.1186/s12943-020-01184-8 PMC7079398 32188489

[35] Krol J , Krol I , Alvarez CPP , Fiscella M , Hierlemann A , Roska B & Filipowicz W (2015) A network comprising short and long noncoding RNAs and RNA helicase controls mouse retina architecture. Nat Commun 6, 7305. 26041499 10.1038/ncomms8305 PMC4468907

[36] Kawahara Y , Zinshteyn B , Sethupathy P , Iizasa H , Hatzigeorgiou AG & Nishikura K (2007) Redirection of silencing targets by adenosine‐to‐inosine editing of miRNAs. Science 315, 1137–1140. 17322061 10.1126/science.1138050 PMC2953418

[37] Wulff B‐E , Sakurai M & Nishikura K (2011) Elucidating the inosinome: global approaches to adenosine‐to‐inosine RNA editing. Nat Rev Genet 12, 81–85. 21173775 10.1038/nrg2915 PMC3075016

[38] Behm M & Öhman M (2016) RNA editing: a contributor to neuronal dynamics in the mammalian brain. Trends Genet 32, 165–175. 26803450 10.1016/j.tig.2015.12.005

[39] Nishikura K (2010) Functions and regulation of RNA editing by ADAR deaminases. Annu Rev Biochem 79, 321–349. 20192758 10.1146/annurev-biochem-060208-105251 PMC2953425

[40] Jain M , Jantsch MF & Licht K (2019) The Editor’s I on disease development. Trends Genet 35, 903–913. 31648814 10.1016/j.tig.2019.09.004

[41] Han L , Diao L , Yu S , Xu X , Li J , Zhang R , Yang Y , Werner HMJ , Eterovic AK , Yuan Y *et al*. (2015) The genomic landscape and clinical relevance of A‐to‐I RNA editing in human cancers. Cancer Cell 28, 515–528. 26439496 10.1016/j.ccell.2015.08.013 PMC4605878

[42] Picardi E , Manzari C , Mastropasqua F , Aiello I , D’Erchia AM & Pesole G (2015) Profiling RNA editing in human tissues: towards the inosinome Atlas. Sci Rep 5, 14941. 26449202 10.1038/srep14941 PMC4598827

[43] Ramaswami G , Zhang R , Piskol R , Keegan LP , Deng P , O'Connell MA & Li JB (2013) Identifying RNA editing sites using RNA sequencing data alone. Nat Methods 10, 128–132. 23291724 10.1038/nmeth.2330 PMC3676881

[44] Gong J , Wu Y , Zhang X , Liao Y , Sibanda VL , Liu W & Guo A‐Y (2014) Comprehensive analysis of human small RNA sequencing data provides insights into expression profiles and miRNA editing. RNA Biol 11, 1375–1385. 25692236 10.1080/15476286.2014.996465 PMC4615373

[45] Alon S , Mor E , Vigneault F , Church GM , Locatelli F , Galeano F , Gallo A , Shomron N & Eisenberg E (2012) Systematic identification of edited microRNAs in the human brain. Genome Res 22, 1533–1540. 22499667 10.1101/gr.131573.111 PMC3409266

[46] Wang Y , Xu X , Yu S , Jeong KJ , Zhou Z , Han L , Tsang YH , Li J , Chen H , Mangala LS *et al*. (2017) Systematic characterization of A‐to‐I RNA editing hotspots in microRNAs across human cancers. Genome Res 27, 1112–1125. 28411194 10.1101/gr.219741.116 PMC5495064

[47] Kume H , Hino K , Galipon J & Ui‐Tei K (2014) A‐to‐I editing in the miRNA seed region regulates target mRNA selection and silencing efficiency. Nucleic Acids Res 42, 10050–10060. 25056317 10.1093/nar/gku662 PMC4150774

[48] Paul D , Sinha AN , Ray A , Lal M , Nayak S , Sharma A , Mehani B , Mukherjee D , Laddha SV , Suri A *et al*. (2017) A‐to‐I editing in human miRNAs is enriched in seed sequence, influenced by sequence contexts and significantly hypoedited in glioblastoma multiforme. Sci Rep 7, 2466. 28550310 10.1038/s41598-017-02397-6 PMC5446428

[49] Kawahara Y , Megraw M , Kreider E , Iizasa H , Valente L , Hatzigeorgiou AG & Nishikura K (2008) Frequency and fate of microRNA editing in human brain. Nucleic Acids Res 36, 5270–5280. 18684997 10.1093/nar/gkn479 PMC2532740

[50] Iizasa H , Wulff B‐E , Alla NR , Maragkakis M , Megraw M , Hatzigeorgiou A , Iwakiri D , Takada K , Wiedmer A , Showe L *et al*. (2010) Editing of Epstein‐Barr virus‐encoded BART6 microRNAs controls their dicer targeting and consequently affects viral latency. J Biol Chem 285, 33358–33370. 20716523 10.1074/jbc.M110.138362 PMC2963350

[51] Yang W , Chendrimada TP , Wang Q , Higuchi M , Seeburg PH , Shiekhattar R & Nishikura K (2006) Modulation of microRNA processing and expression through RNA editing by ADAR deaminases. Nat Struct Mol Biol 13, 13–21. 16369484 10.1038/nsmb1041 PMC2950615

[52] Quinones‐Valdez G , Tran SS , Jun H , Bahn JH , Yang E‐W , Zhan L , Brümmer A , Wei X , Van Nostrand EL , Pratt GA *et al*. (2019) Regulation of RNA editing by RNA‐binding proteins in human cells. Commun Biol 2, 19. 30652130 10.1038/s42003-018-0271-8 PMC6331435

[53] Tariq A , Garncarz W , Handl C , Balik A , Pusch O & Jantsch MF (2013) RNA‐interacting proteins act as site‐specific repressors of ADAR2‐mediated RNA editing and fluctuate upon neuronal stimulation. Nucleic Acids Res 41, 2581–2593. 23275536 10.1093/nar/gks1353 PMC3575830

[54] Choudhury Y , Tay FC , Lam DH , Sandanaraj E , Tang C , Ang B‐T & Wang S (2012) Attenuated adenosine‐to‐inosine editing of microRNA‐376a* promotes invasiveness of glioblastoma cells. J Clin Invest 122, 4059–4076. 23093778 10.1172/JCI62925 PMC3484441

[55] da Silva S , Carolina J , Rocío S & Busturia A (2018) Epigenetic and non‐epigenetic functions of the RYBP protein in development and disease. Mech Ageing Dev 174, 111–120. 29665352 10.1016/j.mad.2018.03.011

[56] Danen‐van Oorschot AAAM , Voskamp P , Seelen MCMJ , van Miltenburg MHAM , Bolk MW , Tait SW , Boesen‐de Cock JGR , Rohn JL , Borst J & Noteborn MHM (2004) Human death effector domain‐associated factor interacts with the viral apoptosis agonist Apoptin and exerts tumor‐preferential cell killing. Cell Death Differ 11, 564–573. 14765135 10.1038/sj.cdd.4401391

[57] Wen MA , Xuan Z , Meng LI , Xiaoli MA , Bingren H , Hong C & Chen D (2016) Proapoptotic RYBP interacts with FANK1 and induces tumor cell apoptosis through the AP‐1 signaling pathway. Cell Signal 28, 779–787. 27060496 10.1016/j.cellsig.2016.03.012

[58] Novak RL & Phillips AC (2008) Adenoviral‐mediated Rybp expression promotes tumor cell‐specific apoptosis. Cancer Gene Ther 15, 713–722. 18551146 10.1038/cgt.2008.25

[59] Stanton SE , Blanck JK , Joseph L & Schreiber‐Agus N (2007) Rybp interacts with Hippi and enhances Hippi‐mediated apoptosis. Apoptosis 12, 2197–2206. 17874297 10.1007/s10495-007-0131-3

[60] Li G , Warden C , Zou Z , Neman J , Krueger JS , Jain A , Jandial R & Chen M (2013) Altered expression of polycomb group genes in glioblastoma multiforme. PLoS One 8, e80970. 24260522 10.1371/journal.pone.0080970 PMC3829908

[61] Herrero MJ & Gitton Y (2018) The untold stories of the speech gene, the FOXP2 cancer gene. Genes & Cancer 9, 11–38. 29725501 10.18632/genesandcancer.169 PMC5931254

[62] Tsui D , Vessey JP , Tomita H , Kaplan DR & Miller FD (2013) FoxP2 regulates neurogenesis during embryonic cortical development. J Neurosci 33, 244–258. 23283338 10.1523/JNEUROSCI.1665-12.2013 PMC6618635

[63] Khan FH , Pandian V , Ramraj S , Natarajan M , Aravindan S , Herman TS & Aravindan N (2015) Acquired genetic alterations in tumor cells dictate the development of high‐risk neuroblastoma and clinical outcomes. BMC Cancer 15, 514. 26159519 10.1186/s12885-015-1463-y PMC4496850

[64] Cohen AL , Holmen SL & Colman H (2013) IDH1 and IDH2 mutations in gliomas. Curr Neurol Neurosci Rep 13, 345. 23532369 10.1007/s11910-013-0345-4 PMC4109985

[65] Franco‐Zorrilla JM , Valli A , Todesco M , Mateos I , Puga MI , Rubio‐Somoza I , Leyva A , Weigel D , García JA & Paz‐Ares J (2007) Target mimicry provides a new mechanism for regulation of microRNA activity. Nat Genet 39, 1033–1037. 17643101 10.1038/ng2079

[66] Chi SW , Zang JB , Mele A & Darnell RB (2009). Argonaute HITS‐CLIP decodes microRNA–mRNA interaction maps. Nature 460, 479–486. 19536157 10.1038/nature08170 PMC2733940

[67] Shan K , Jiang Q , Wang X‐Q , Wang Y‐N‐Z , Yang H , Yao M‐D , Liu C , Li X‐M , Yao J , Liu B *et al*. (2016) Role of long non‐coding RNA‐RNCR3 in atherosclerosis‐related vascular dysfunction. Cell Death Dis 7, e2248. 27253412 10.1038/cddis.2016.145 PMC5143375

[68] Terreri S , Durso M , Colonna V , Romanelli A , Terracciano D , Ferro M , Perdonà S , Castaldo L , Febbraio F , de Nigris F *et al*. (2016) New cross‐talk layer between ultraconserved non‐coding RNAs, microRNAs and polycomb protein YY1 in bladder cancer. Genes 7, 127. 10.3390/genes7120127 PMC5192503 27983635

[69] Francesco I , Knijnenburg TA , Vis DJ , Bignell GR , Menden MP , Michael S , Nanne A , Emanuel G , Syd B , Howard L *et al*. (2016) A landscape of pharmacogenomic interactions in cancer. Cell 166, 740–754. 27397505 10.1016/j.cell.2016.06.017 PMC4967469

