# Supplementary material for: The transcribed ultraconserved region uc.160+ enhances processing and A‐to‐I editing of the miR‐376 cluster: hypermethylation improves glioma prognosis
Source: Mol Oncol. 2021 Nov 3;16(3):648–64. doi: 10.1002/1878-0261.13121 (PMC8807354; doi:10.1002/1878-0261.13121)
Supplement: Supplementary file 1 — Fig. S1. Methylation status of uc.160+ in normal tissues, expression patterns and coding petential. Fig. S2. Expression of miR‐376 cluster membres in tissues and cell lines, and in vitro modulation of their processing by uc.160+. Fig. S3. ADAR1 and 2 levels in glioma cell lines, and disruption of miR‐376 function. Fig. S4. uc.160+ CpG island hypermethylation in high‐grade gliomas and its association with clinical outcome. Table S1. Oligos used in this work. [file MOL2-16-648-s001.docx]

**SUPPLEMENTAL INFORMATION (Soler et al).**

This file contains:

Supplementary Figures and Legends (Supplementary Figures S1-S4)

Supplementary Table (Table S1).

**Supplementary Figure S1.** Methylation status of *uc.160+* in normal tissues, expression patterns and coding potential. **(A)** Percentage of *uc.160+* methylated samples in the TCGA panel of normal tissues. **(B)** RT-PCR end-point from total and nuclear RNA of U-87 MG to assess the miTranscriptome sequence (DNA was used as a positive control). Reverse transcription was carried out with random primers (+RT, with reverse transcriptase; –RT, without reverse transcriptase) and PCR was conducted with specific forward (F1) and reverse (R1) primers. **(C)** Coding potential of the *uc.160+* transcript, as annotated in MiTranscriptome. Spliced and unspliced products were analyzed. For comparison, results corresponding to the noncoding *H19* and *HOTAIR* RNAs, and the protein-coding *GAPDH* transcripts are shown.

**Supplementary Figure S2.** Expression of *miR-376* cluster members in tissues and cell lines, and *in vitro* modulation of their processing by *uc.160+*. **(A)** Expression of *miR-376* family members in normal samples, classified by tissue. Values from raw expression, taken from <https://ccb-web.cs.uni-saarland.de/tissueatlas/patterns>. **(B)** Assessment of endogenous levels of mature *miR-376a*, *miR-376b* and *miR-376c* by RT-qPCR in U-87 MG and KS-1 cell lines. **(C)** Diagram to illustrate the mutant sequences used in binding assays and transfection experiments. The 12-nucleotide complementary site between *uc.160+* and *pri-miR-376* family was completely (mut12) or partially (mut5) changed. Compensatory mutations on pri-miRNAs were also introduced for mut5. Figure created with BioRender.com. **(D)** Electrophoretic mobility shift assay (EMSA) with biotin-labeled *uc.160+ mut5* RNA and increasing levels of unlabeled *pri-miRNA-376a-1/a-2/b/c* *mut5* (0.5-1-2-4 pmols). **(E)** Electrophoretic mobility shift assay (EMSA) with biotin-labeled *pri-miRNA-376a-1/a-2/c* *mut5* RNA and increasing levels of unlabeled *uc.160+, mut5* or *mut12* (1-2-4 pmols). **(F)** *Pri-miR-376* in vitro processing assays in the presence of increasing concentrations of *uc.160+.* **(G)** Diagrams illustrating the region of complementarity between *uc.160+* and *pri-mir-376-a1, a2* and *b.* Stem-loop structure is depicted according to (32). The complementarity sequences are highlighted in green, and the CNNC regions are boxed in red. The mature miRNA sequences are shown in blue. Black arrows indicate Drosha cropping sites.

**Figure S3.** ADAR1 and 2 levels in glioma cell lines, and disruption of *miR-376* function. **(A)** Western blot to detect ADAR1 and ADAR2 protein levels in KS-1 and U-87 MG cell lines upon transient transfection of *uc.160+*. Control-transfected cells (empty vector, EV) are shown for comparison. **(B)** Western blot analysis of ADAR1 and ADAR2 proteins in a panel of glioma cell lines. The HEK 293T cell line was used as a control. **(C)** Validation of antagomiR function in KS-1 and U-87 MG cells was conducted by means of RYBP and FOXP2 protein level analysis by western blot. Both miRNA mimics and antagomiRs were transfected in parallel with their respective controls (scr-miR and scr-antagomiR). **(D)** *Left*, diagram to illustrate the CRISPR-mediated strategy to knockout the *miR-376* cluster. PCR fragment sizes for the original and the predicted edited regions are indicated. Figure created with BioRender.com. *Middle*, end-point PCR on genomic DNA from control (mock) and CRISPR-edited (sg1+sg2) KS-1 cells. *Right*, RT-qPCR analysis of the endogenous levels of *miR-376a*, *miR-376b* and *miR-376c* in control or edited KS-1 cells. **(E)** Western blot to assess RYBP and FOXP2 protein levels upon *uc.160+* overexpression in control or edited KS-1 cells.

**Figure S4.** *uc.160+* CpG island hypermethylation in high-grade gliomas and its association with clinical outcome. **(A)** Kaplan–Meier analysis of OS of high-grade glioma in wild-type *IDH1* patients with respect to *uc.160+* CpG island methylation status. Probabilities (*p*) are those associated with log-rank tests. Results of univariate Cox regression are presented as the hazard ratio (HR) and 95% confidence interval (CI). The data available for all GBM-mutant *IDH1* patients indicated that they were all alive. **(B, C)** Forest plot of the multivariate Cox regression for clinical outcome in the TCGA glioma cohorts analyzed according to *uc.160+* methylation status (B, all-grade patients, and C, high-grade glioma). Probabilities (*p*) and 95% confidence intervals (CI) correspond to the hazard ratios (HR) associated with OS. Significant covariates were considered to be independent prognostic factors (*, *p* < 0.05; **, *p* < 0.01; ***, *p* < 0.001).

**Table S1**. Oligos used in this work.

| **Name** | **Sequence 5' - 3'** | **Experiment** |
| --- | --- | --- |
| uc.160 BamHI Fw | TCGGGATCCAGTAAATGAGGCGAGTGTGC | Cloning |
| uc.160 EcoRV Rv | TCGGATATCAGCATCCTTAATATTTCCTTCC | Cloning |
| uc.160 mutant 12 Fw | GCAAGAGCATTATTTACGGGTCGCAGCTACATCTGTGATTTAAAAC | Mutant construction |
| uc.160 mutant 12 Rv | GTTTTAAATCACAGATGTAGCTGCGACCCGTAAATAATGCTCTTGC | Mutant construction |
| uc.160 mutant 5 Fw | GCAAGAGCATTATTTACGGTGATACTCTACATC | Mutant construction |
| uc.160 mutant 5 Rv | GATGTAGAGTATCACCGTAAATAATGCTCTTGC | Mutant construction |
| Pri376c mut 5 Fw | GTTTTCAGTATCACCGTATGCTTGGAAACATTC | Mutant construction |
| Pri376c mut 5 Rv | GAATGTTTCCAAGCATACGGTGATACTGAAAAC | Mutant construction |
| Pri376a-1 mut 5 Fw | CGTTTTCAGTATCACCGTATGCTTTGAAAACCTC | Mutant construction |
| Pri376a-1 mut 5 Rv | GAGGTTTTCAAAGCATACGGTGATACTGAAAACG | Mutant construction |
| Pri376a-2 mut 5 Fw | CGTTTTCAGTATCACCGTATGCCTTGGGATCATG | Mutant construction |
| Pri376a-2 mut 5 Rv | CATGATCCCAAGGCATACGGTGATACTGAAAACG | Mutant construction |
| Pri376b mut 5 Fw | GTTTTCAGTATCACCGTATGCTTTGGAAATG | Mutant construction |
| Pri376b mut 5 Rv | CATTTCCAAAGCATACGGTGATACTGAAAAC | Mutant construction |
| Scr (Negative Control NC) sense | UUCUCCGAACGUGUCACGUTT | transfection of mimics |
| Scr (Negative Control NC) antisense | ACGUGACACGUUCGGAGAATT | transfection of mimics |
| hsa-miR-376c-3p MIMAT0000720 sense | AACAUAGAGGAAAUUCCACGU | transfection of mimics |
| hsa-miR-376c-3p MIMAT0000720 antisense | GUGGAAUUUCCUCUAUGUUUU | transfection of mimics |
| hsa-miR-376a-3p MIMAT0000729 sense | AUCAUAGAGGAAAAUCCACGU | transfection of mimics |
| hsa-miR-376a-3p MIMAT0000729 antisense | GUGGAUUUUCCUCUAUGAUUU | transfection of mimics |
| hsa-miR-376c-3p Edited form sense | AACAUIGAGGAAAUUCCACGU | transfection of mimics |
| hsa-miR-376c-3p Edited form antisense | GUGGAAUUUCCUCCAUGUUUU | transfection of mimics |
| hsa-miR-376a-3p Edited form sense | AUCAUIGAGGAAAAUCCACGU | transfection of mimics |
| hsa-miR-376a-3p Edited form antisense | GUGGAUUUUCCUCCAUGAUUU | transfection of mimics |
| pri-miR376a2-c editing Rv | TTCAGTCCAGCCATGATCCC | Synthesis of cDNA for editing analysis |
| pri-miR376a1-b editing Rv | TGGCGACTTCACGTCCTCCGA | Synthesis of cDNA for editing analysis |
| pri-miR376a2-c Fw | CCCCTGCCGACTGCACCTAT | PCR for editing analysis |
| pri-miR376a2-c Rv | TTCAGTCCAGCCATGATCCC | PCR for editing analysis |
| pri-miR376a1-b Fw | TGTCCTTTCCAGAGCCCAGTCC | PCR for editing analysis |
| pri-miR376a1-b Rv | TGGCGACTTCACGTCCTCCGA | PCR for editing analysis |
| FOXP2 Fw | GCGTCAGGGACTCATCTCC | RT-qPCR |
| FOXP2 Rv | GAGGTCTAGCCCTCCATGTTTA | RT-qPCR |
| RYBP Fw | GGGTTTTGGGATTGTAGCGTC | RT-qPCR |
| RYBP Rv | GTGCCACCAGCTGAGAATTG | RT-qPCR |
| PPiA Fw | ATGGTCAACCCCACCGTGT | RT-qPCR |
| PPiA Rv | TCTGCTGTCTTTGGGACCTTG | RT-qPCR |
| HPRT1 Fw | TGACACTGGCAAAACAATGCA | RT-qPCR |
| HPRT1 Rv | GGTCCTTTTCACCAGCAAGCT | RT-qPCR |
| GUSB Fw | TGGTTGGAGAGCTCATTTGGA | RT-qPCR |
| GUSB Rv | GCACTCTCGTCGGTGACTGTT | RT-qPCR |
| GAPDH Fw | TCTTCTTTTGCGTCGCCAG | RT-qPCR |
| GAPDH Rv | AGCCCCAGCCTTCTCCA | RT-qPCR |
| RNU6B Fw | CTCGCTTCGGCAGCACA | RT-qPCR |
| RNU6B Rv | AACGCTTCACGAATTTGCGT | RT-qPCR |
| hsa-miR-376a-3p miRCURY LNA miRNA PCR Assay | GeneGlobe ID. - YP00204508 | RT-qPCR |
| hsa-miR-376b-3p miRCURY LNA miRNA PCR Assay | GeneGlobe ID**. -**YP00204218 | RT-qPCR |
| hsa-miR-376c-3p miRCURY LNA miRNA PCR Assay | GeneGlobe ID. - YP00204442 | RT-qPCR |
| U6 snRNA(hsa, mmu) miRCURY LNA miRNA PCR | GeneGlobe ID. - YP00203907 | RT-qPCR |
| hsa-miR-191-5p miRCURY LNA miRNA PCR Assay | GeneGlobe ID. - YP0024306 | RT-qPCR |
| hsa-miR-423-3p miRCURY LNA miRNA PCR Assay | GeneGlobe ID - YP00204488 | RT-qPCR |
| uc.160-miT Fw1 | CTCCGTTCTCATCCTGCAC | PCR |
| uc.160-miT Rv1 | CCATCTTCTGCCCAACTCAT | PCR |
| pri376cfor | GGTGAATCCCAGAACCTAAGC | pri-miRNA template construction |
| pri376crev | CATATGGCACGAATTCAGCA | pri-miRNA template construction |
| pri376a2for | GAGACCCCTCTCGCAAGC | pri-miRNA template construction |
| pri376a2rev | GGGGCTTCTAAAGGTGATGG | pri-miRNA template construction |
| pri376bfor | CACCTTTAGAAGCCCCCTTT | pri-miRNA template construction |
| pri376brev | AAGAACTGGCACCACGAGAC | pri-miRNA template construction |
| pri376a1for | TCTGAATGAACGGGGTATGC | pri-miRNA template construction |
| pri376a1rev | GCAGTGAGCAAACAGCAGAG | pri-miRNA template construction |
| sgRNA-1 BbsI Fw | CACCgcactttgcgagtcccacgt | Cloning of CRISPR sgRNA1 |
| sgRNA-1 BbsI Rv | AAACacgtgggactcgcaaagtgc | Cloning of CRISPR sgRNA1 |
| sgRNA-2 BbsI Fw | CACCgatggtgagagcagcacaccg | Cloning of CRISPR sgRNA2 |
| sgRNA-2 BbsI Rv | AAACcggtgtgctgctctcaccatC | Cloning of CRISPR sgRNA2 |
| CRISPR-376 Fw | TCTTGACCATGAAGCCTCCA | PCR for CRISPR analysis |
| CRISPR-376 Rv | TGATGGCAGTGACAGGAAGG | PCR for CRISPR analysis |
| Pri-376c for1 | TGCTTAGGTTCATGCTTTCCAG | Detection of pri-miRNA by RT-qPCR |
| Pri-376c rev1 | TGGTTTCAGTCCAGGAATGTT | Detection of pri-miRNA by RT-qPCR |
